# Comparative Proteomic Analysis Reveals New Insights Into the Common and Specific Metabolic Regulation of the Diatom *Skeletonema dohrnii* to the Silicate and Temperature Availability

**DOI:** 10.3389/fpls.2020.578915

**Published:** 2020-11-05

**Authors:** Satheeswaran Thangaraj, Mario Giordano, Jun Sun

**Affiliations:** ^1^College of Marine Science and Technology, China University of Geosciences (Wuhan), Wuhan, China; ^2^Dipartimento di Scienze della Vita e dell’Ambiente, Università Politecnica delle Marche, Ancona, Italy

**Keywords:** silicate, temperature, diatom, iTRAQ, ribosome metabolism, photosynthesis, comparative proteomics, carbon metabolism

## Abstract

Silicate (Si) and temperature are essential drivers for diatom growth and development in the ocean. Response of diatoms to these particular stress has been investigated; however, their common and specific responses to regulate intracellular development and growth are not known. Here, we investigated the combination of physiological characteristics and comparative proteomics of the diatom *Skeletonema dohrnii* grown in silicate- and temperature-limited conditions. Results show that cell carbon and lipid quotas were higher at lower-temperature cells, whereas cellular phosphate was higher in cells grown with lower Si. In silicate-limited cells, nitrate transporters were downregulated and resulted in lower nitrate assimilation, whereas the phosphate transporters and its assimilation were reduced in lower-temperature conditions. In photosynthesis, lower silicate caused impact in the linear electron flow and NADPH production, whereas cycling electron transport and ATP production were affected by the lower temperature. Concerning cell cycle, imbalances in the translation process were observed in lower-silicate cells, whereas impact in the transcription mechanism was observed in lower-temperature cells. However, proteins associated with carbon fixation and photorespiration were downregulated in both stress conditions, while the carbohydrate and lipid synthesis proteins were upregulated. Our results showed new insights into the common and specific responses on the proteome and physiology of *S. dohrnii* to silicate and temperature limitation, providing particular nutrient (Si)- and temperature-dependent mechanisms in diatoms.

## Introduction

Diatoms are key ecological players in the contemporary ocean, responsible for 40% of oceanic primary production (Falkowski et al., 1998); they contribute extensively to the global carbon cycle (Martin et al., 2011). They are possibly the most abundant silicified organisms on Earth (Shrestha et al., 2012) due to their silicon cell wall (Allen et al., 2011) believed to be the reason for their ecological success (Hamm et al., 2003). Silicate is essential for diatoms not only because of their cell walls but also because of its requirement for diatom metabolism similar to other nutrients, i.e., nitrate, phosphate, and iron (Thangaraj et al., 2019). Temperature has a greater influence on algal metabolism (Bermudez et al., 2015), including diatoms (Depauw et al., 2012), because of its involvement in a high number of metabolic processes and enzymatic reactions. Recent reports demonstrated that changes in the temperature could alter the overall transcriptome (Liang et al., 2019) and proteome profile of diatom (Dong et al., 2016), leading to metabolic imbalances in amino acid biosynthesis and photosynthesis metabolism.

Silicon transporters (SITs) have recently been characterized in diatoms (Shrestha and Hildebrand, 2015); they are regulated by Si bioavailability, resulting in changes in gene expression and nutrient assimilation (Smith et al., 2016). Nutrient limitation could suppress the photosynthesis mechanism of phytoplankton depending on temperature (Maranon et al., 2018), showing that photosynthesis proteins and associated electron transport in diatom could change their nature with Si bioavailability (Thangaraj et al., 2019). In addition, transcriptome (Brembu et al., 2017) and proteome studies (Chen et al., 2018) on diatom’s carbon metabolism have shown that changes in Si availability could decrease carbon fixation with simulation increase in the lipid accumulation for the acclimation process (Smith et al., 2016). The interrelation between amino acid biosynthesis and associated protein processing of ribosomal assembly in eukaryotes has been demonstrated (Lott et al., 2013) to alter the growth rate and cellular development (Zhou et al., 2015) under stressful conditions. Although cell cycle-related proteins were characterized in diatom response to Si deficiency (Du et al., 2014), it has not been explored whether the interconnection of amino acid biosynthesis and associated protein processing would alter growth and cellular development.

Temperature is essential for diatoms’ photosynthetic process and enzymatic reactions (Huysman et al., 2013). In diatoms, each species has its own characteristic response to temperature fluctuation (Boyd and Hutchins, 2012) to evolve a sophisticated cellular mechanism for their ecological success (Brunet and Lavaud, 2010). Nevertheless, current understanding of the light-driven process in marine diatoms is still limited at the molecular level (Depauw et al., 2012), especially at lower temperatures (night) where algae consume 22% of their biomass (Edmundson and Huesemann, 2015) and exhibit NPQ as an important photo-protective process (Jallet et al., 2016) to manage their cellular energy and dissipation. The transcriptome response of diatoms has shown that, under lower temperatures, amino acid biosynthesis and ribosome complex altered their functions (Liang et al., 2019). In contrast, higher temperatures stimulated changes in the diatom’s photosynthesis and associated electron transport along with the light-harvesting complex (Dong et al., 2016). Maranon et al. (2018) reported that nutrient uptake at the molecular level can be influenced by temperature limitation, although it is the consequence of the interplay of nutrient availability and transporters. The transcriptome (Liang et al., 2019; Thangaraj and Sun, 2020a) and proteomic changes (Dong et al., 2016) of a diatom’s response to changing temperature showed that temperature is the dominant factor altering gene expression in cell cycle progress and protein synthesis for cellular development and growth. Also, metabolic changes in multiple diatoms revealed that changes in carbon metabolism could be temperature-dependent, determining decreased photosynthesis and carbon fixation with increased lipid accumulation for their acclimation process (Liang et al., 2019).

The ecological success of diatoms has determined the emergence of an interest in the understanding of the regulation of their proteome in response to a variety of environmental parameters: nitrate (Jian et al., 2017), phosphate (Dyhrman et al., 2012), silicate (Du et al., 2014), iron (Nunn et al., 2013), and availability of temperature (Dong et al., 2016). Although the outcome of these studies has shown significant metabolic regulation or proteome exposed to specific factors, there is a lack of information on the interplay of different environmental effectors and their cumulative effect on cellular growth and development. In order to address this matter, iTRAQ-based comparative proteomics was applied in this study to compare the proteome profile of the diatom *Skeletonema dohrnii* grown in silicate and temperature limitation. Temperature was chosen because of it influenced metabolism in a non-specific mode (although it may elicit specific responses for the amelioration of stresses linked to certain functions), whereas silicon is specifically related the construction and maintenance of the involucral system of the cell.

## Materials and Methods

### Sampling and Culture Condition

This study was conducted using ecologically important diatom *S. dohrnii*, isolated from the Yellow Sea. The isolated strain was maintained in Artificial Seawater Media (ASW) (Sunda et al., 2005), at 25°C with an irradiance of 100–120 μmol photons m^–2^ s^–1^, at a 12:12-h light–dark cycle (Thangaraj and Sun, 2020b). For the experiment, cultures were grown in two different temperatures (control 25°C and lower temperature 15°C) and silicate concentrations (control 1 ml/L and lower silicate 0.2 ml/L), respectively, with the abovementioned conditions. The final concertation of used silicate stock solution in this study was 229.71 μM/ml. To prevent the extra silicon utilization of *S. dohrnii* during the experiment, cells were grown in Nalgene, polycarbonate bottles for the silicate treatment. Both silicate- and temperature-limited cells were harvested during mid-exponential growth (day 4) filtering through 2-mm pore-size membrane filter for the cellular elements (C, N, P, and Si), macromolecular components (carbohydrate, lipids, and protein), and quantitative proteomic analysis.

### Measurements of Growth Rate, Nutrient Analysis, and Cell Constituents

The reproducibility of each condition was checked by using three independent triplicate samples, for cell growth, nutrients analysis, cellular elements, and biochemical properties. The growth rate μ (day^–1^) in this study was calculated as follows:

μ=1n(N:tN)0/t

where, *N*_0_ and *N*_*t*_ are at the end and start of the exponential phase of growth, respectively, and *t* is the duration of the exponential growth phase. The concentration of nutrients (N, P, and Si) in each medium of culture was measured following continuous flow analysis (CFASAN Plus/Skalar Analytik, Germany). To determine the cellular elements (C, N, P, and Si), culture aliquots containing about 2 × 10^7^ cells were collected and dried in an oven at 65°C for 2 days, prior to pulverization in a tissue layer (Elementar, Germany). For N, P, and Si, the pulverized samples were transferred into a tinfoil cup and analyzed with a photometric auto-analyzer (CFASAN Plus/Skalar Analytic, Germany) and EL cube (Elementar, Germany) following the protocol described by Boyd et al. (2015); Chen et al. (2018). For cellular elements, the total carbohydrate was analyzed using the anthrone method (Guerra et al., 2013); total lipids were measured according to protocol described by Yoneda et al. (2018) and determined gravimetrically using a microanalytical balance. The total proteins were determined following the Folin-Phenol method of Lowry et al. (1951). Chlorophyll *a* was extracted in 90% acetone, at 4°C for 24 h in the dark and quantified spectrophotometrically using the equation in Jeffrey and Humphrey (1975). All measurements were carried out on three biological replicates; statistical significance was assessed by *t*-test.

### Protein Extraction, Preparation, and Digestion

One liter of culture from each sample was collected through 2 μM filter membrane; then, subsequently, samples were suspended in 10 ml of medium into 15-ml centrifuge tubes for protein preparation (Du et al., 2014). The resulting cell pellets were then suspended in lysis buffer 3 (8 M Urea, 40 mM Tris-HCl or TEAB with 1 mM PMSF, 2 mM EDTA, and 10 mM DTT, pH 8.5). The mixture of samples was placed into a tissue lyser for 2 min at 50 Hz to cell lysis and then centrifuged at 25,000*g* for 20 min at 4°C. The supernatant was then transferred into a new tube; samples were reduced with 10 mM dithiothreitol (DTT) at 56°C for 1 h and alkylated by 55 mM iodoacetamide (IAM) in the dark at room temperature for 45 min to block the cysteine residues of the proteins. Following centrifugation (25,000*g* for 20 min at 4°C), the supernatant containing proteins was quantified by the Bradford assay method (Kruger, 2009). The protein solution (100 μg) with 8 M urea was diluted four times with 100 mM TEAB. Trypsin Gold (Promega, Madison, WI, United States) was used for the protein digestion with a ratio of trypsin = 40:1 at 37°C overnight. After trypsin digestion, peptides were desalted with a Strata X C18 column (Phenomenex) and vacuum-dried according to the manufacturer’s protocol for 8-plex iTRAQ (Applied Biosystems, Foster City, CA, United States). Each treatment was made up of two (control) or three (treated with lower Si or lower temperature) biological replicates. Briefly, peptides were labeled with iTRAQ reagents 113 and 115 for control samples; 114, 116, and 118 for lower-silicate samples; and 117, 119, and 121 for lower-temperature samples, and then pooled and dried by vacuum centrifugation.

### Analytical Procedure and Peptide Labeling

The labeled peptide blends were pooled and dried through vacuum centrifugation and fractionated. All solvents used for high-performance liquid chromatography (HPLC) were HPLC grade (Sigma-Aldrich), and the H_2_O was Millipore Milli-Q PF filtered. The peptides were separated on a Shimadzu LC-20AB HPLC Pump system coupled with a high-pH reverse-phase column (Gemini C_18_ 5 μM, 4.6 × 250 mm). The peptides were reconstituted to HPLC separation with the following mobile phase: (A) 5% ACN, (B) 95% H_2_O (adjusted pH to 9.8 with 2 ml of NH_3_), sample input and acquisition: 2 ml/min flow rate and 1 ml/min injection volume. Crude peptide compound elution was monitored by measuring UV absorbance at 214 nm, and the 40 fractions were collected every 1 min. All the eluted peptides were combined as 20 fractions and vacuum-dried for further process. Furthermore, each fraction was resuspended in buffer A (2% ACN and 0.1% formic acid in H_2_O) and then centrifuged at 20,000*g* for 10 min and independently subjected to HPLC separation (LC-20AD nano-HPLC instrument, Shimadzu, Kyoto, Japan) using C_18_ column (inner diameter, 75 μm). Sample input and acquisition: 300 nl/min flow rate and 1 μl injection volume for 8 min; the 35-min gradient was run at 300 nl/min starting from 8 to 35% of buffer B (2% H_2_O and 0.1% FA in ACN), followed by a 5-min linear gradient to 80% solution B, maintenance at 80% solution B for 4 min, and return to 5% in 0.1 min and equilibrated for 10 min.

### LC-MS/MS Proteomic Analysis

Liquid chromatography and mass spectrometry (LC-MS) analysis of diatom peptide was performed on LC-20AD (Shimadzu, Kyoto, Japan) using C_18_ column (size, 75 μm). The LC-MS data were acquired in positive ion mode with a selected mass range of 350–1500 *m/z*. Based on the intensity in the MS1 survey, as many as 30 production scans were collected if beyond a threshold of 120 counts per second (counts/s) and charge state 2+ to 5+ dynamic exclusion was set for 1/2 of peak width (12 s). For MS data acquisition, the collision energy was adjusted to all precursor ions for collision-induced dissociation, and the Q2 transmission window for 100 Da was 100%. The greatest extents of the iTRAQ reporter ions imitate the relative abundance of the proteins in the samples. A TripleTOF 5600 mass spectrometer with high mass accuracy resolution (less than 2 ppm) was used in this study for peptide identification. Other identification parameters used included the following: fragment mass tolerance: ± 0.1 Da; mass values: monoisotopic; variable modifications: Gln- > pyro-Glu (N-term Q), oxidation (M), iTRAQ8plex (Y); peptide mass tolerance: 0.05 Da; max missed cleavages: 1; fixed modifications: carbamidomethyl (C), iTRAQ8plex (N-term), iTRAQ8plex (K); other parameters: default.

### Proteomic Data Analysis

All the mass spectral data were processed using the Proteo Wizard software msConvert with default parameters for generating peak list, and the data alignment was performed with Analyst QS 2.0 software (Applied Biosystems/MDS SCIEX). Further, protein identification and quantification were achieved using Mascot 2.3.02 (Matrix Science, London, United Kingdom) (Charbonneau et al., 2007). For iTRAQ quantification, the peptide for quantification was automatically selected by the algorithm to calculate the reporter peak area (using default parameters in the Mascot Software package). The acquired data were auto bias-corrected to get rid of any differences imparted due to the unequal mixing during combining differently labeled samples. Proteins with a 1.2-fold change between each different sample and a *p* value of statistical evaluation less than 0.05 were determined as differentially expressed proteins (DEPs). The Student’s *t*-test was performed using the mascot 2.3.02 software. Briefly, a protein ratio is reported in boldface if it is significantly different from unity. The comparison test is:

$$\left|X-\_\right|\,\_\,t*\frac{S}{\sqrt{N}}$$

If this dissimilarity is real, then there is no important difference at the stated sureness level. Further, *N* is the number of peptide ratios, *S* is the standard deviation, and *X* is the mean of the peptide ratios, with both numbers calculated in log space. The real value of the ratio, μ, is 0 in log space. *t* is Student’s *t* for *N* - 1 degrees of freedom and a two-sided confidence level of 95%.

### Functional Annotation

The COG (Cluster of Orthologous Groups of proteins) and then GO (Gene Ontology) analyses were performed according to the method reported by Unwin (2010). The identification of differentially regulated proteins in GO terms was carried out using the following formula:

$$P=1-{\text{â}}_{i=0}^{m-1}\,\frac{\left(\frac{M}{i}\right)\,\left(\frac{N-M}{n-i}\right)}{\left(\frac{N}{n}\right)}$$

Where *N* is the number of all proteins with GO annotation information, *n* is the number of the differentially regulated proteins with GO annotation information, *M* is the number of proteins with a given GO term annotation, and *m* is the number of the differentially regulated proteins with a given GO term annotation. The GO terms with a *p* value of less than 0.05 were considered as enriched GO terms by the silicate- and temperature-responsive proteins. Proteins with twofold changes between each sample and a *p* value of less than 0.05 were considered as differentially expressed. The metabolic pathway analysis of DEPs was conducted according to the KEGG Pathway Database (Kanehisa et al., 2016).

## Results

### Physiological and Biochemical Responses

**FIGURE 1:**
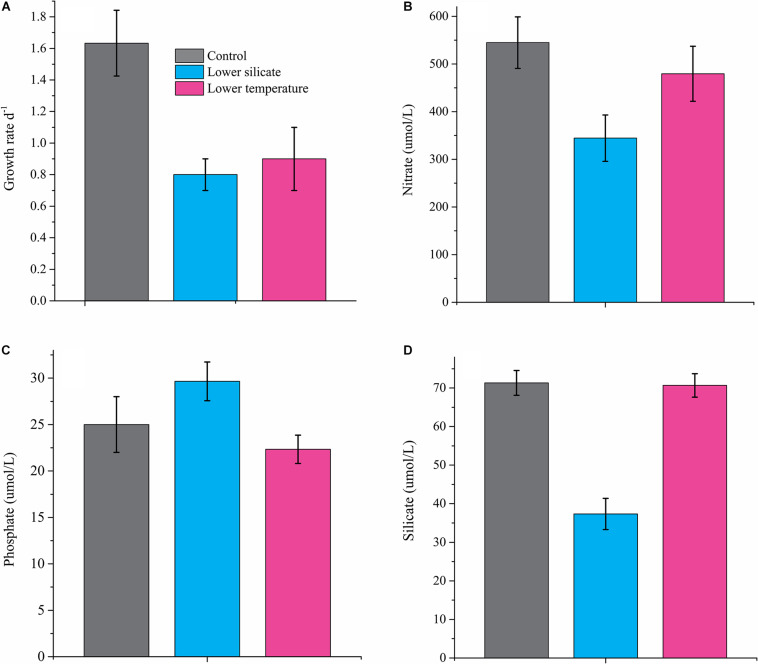
The specific growth rate of *S. dohrnii* grown under normal conditions, silicate and temperature limitation **(A)**, and external nutrient concentration, nitrate **(B)**, phosphate **(C)**, and silicate **(D)** in culture media. Error bars represent the standard deviation of the means (*n* = 3).

Both lower Si and temperature resulted in significant changes in the growth rate of the diatom *S. dohrnii* (Figure 1A), showing 50% reduction in cells grown with lower Si (0.8 ± 0.1) and 45% reduction at lower temperatures (0.9 ± 0.2) compared with the normal condition (1.6 ± 0.2). The concentration of nutrients (N, P, and Si) in the culture media of all the conditions is shown in Figures 1B–D. Results show that compared with the normal culture condition, the concentration of N and Si is lower in Si-limited cells, whereas P was lower in cells limited by temperature.

**FIGURE 2:**
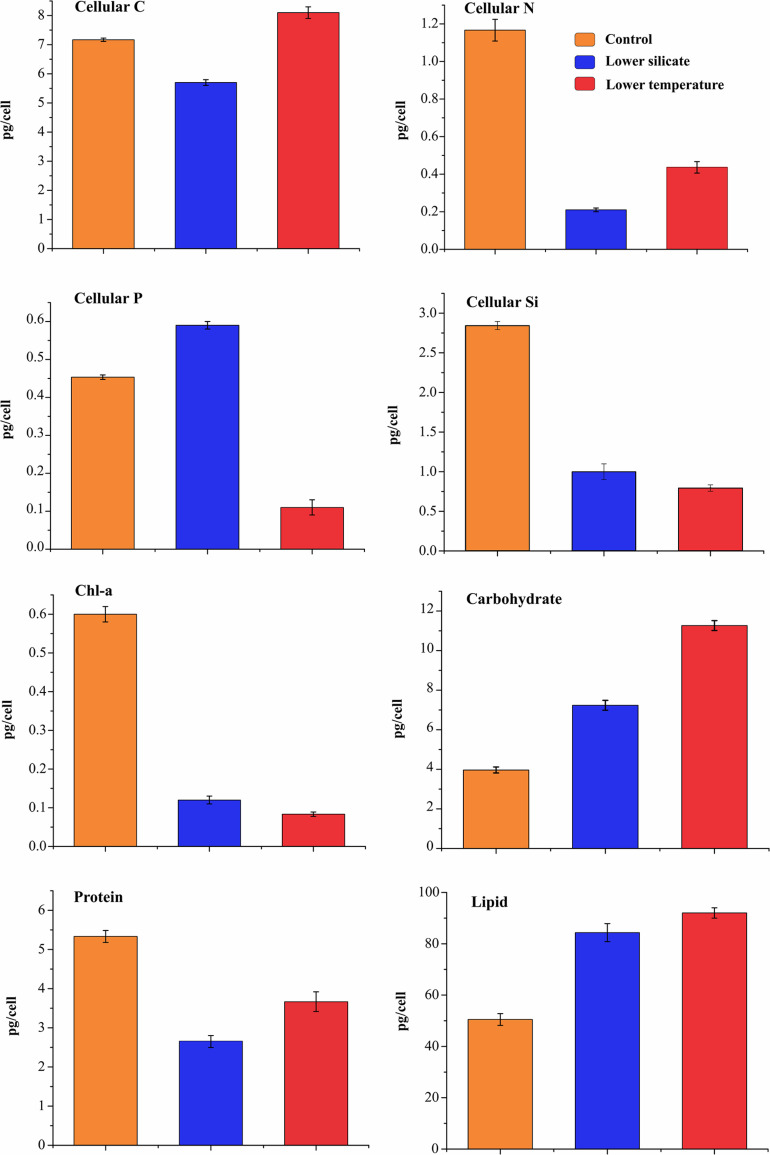
Cellular elements and macromolecular component composition in *S. dohrnii* grown under control, silicate-, and temperature-limited conditions. The error bars represent the standard deviation of the means (*n* = 3).

The cell quotas of the main macronutrients and organic pools are shown in Figure 2, for cells grown at lower-silicate, lower-temperature, and control conditions. In the Si treatment, C quota was 20% lower than in the control cells; the N and Si cell quotas were much lower than in the controls, whereas P content was higher. In lower-temperature treatment, C quota was 12% higher than in the Si treatment and N was lower than in the control but was double as compared to Si-limited cells. The P cell quota was much lower in the temperature treatment cells than in both control and Si treatment, and Si cell quotas were the lowest in temperature treatment with respect to organic pools. Compared with control cells, both silicate and temperature treatment cells have increased the size of carbohydrates and lipid pools but decreased protein synthesis.

### Overview of Quantitative Proteomics

**FIGURE 3:**
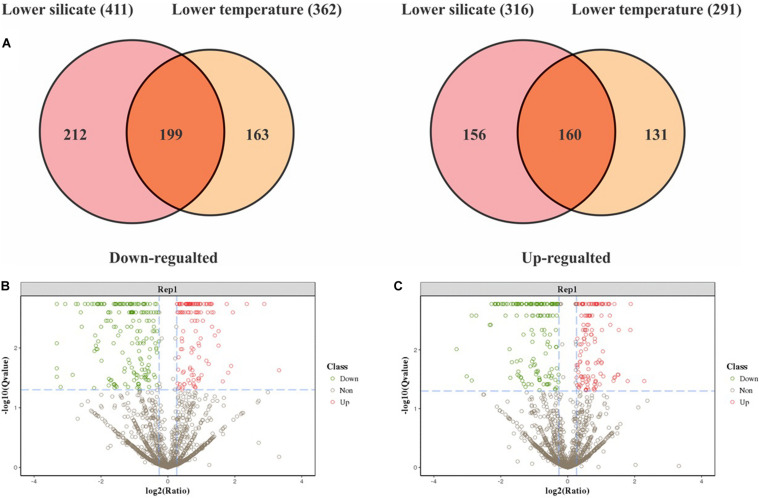
The Venn diagram depicts common and specific differentially expressed proteomic responses between silicate and temperature limitation **(A)**, and volcano plots of differentially expressed proteins in lower-silicate cells **(B)**, and lower-temperature cells **(C)**.

In total, 343,191 spectra were collected, corresponding to 3479 peptides and 1772 proteins that were identified with 1% of FDR. Detailed information—accession numbers, protein descriptions, unique peptide, spectrum, *p*-Value, and *q*-Value with repeatability analysis between the samples—is given in Supplementary Table 1. Protein abundances changed by at least twofold with *p* values lower than 0.05; 1380 proteins were differentially expressed in this study between silicate and temperature limitation (Supplementary Table 2). In lower-silicate cells, 411 proteins were downregulated and 316 were upregulated, and in the lower-temperature cells, 291 proteins were upregulated and 362 were downregulated. Among them, 359 proteins were commonly expressed in both conditions, of which 199 were downregulated and 160 were upregulated (Figure 3A). These DEPs were then plotted using the volcano plot analysis, shown in Figures 3B,C.

### Functional Classification of COG and GO and KEGG Pathway Enrichment Analysis

**FIGURE 4:**
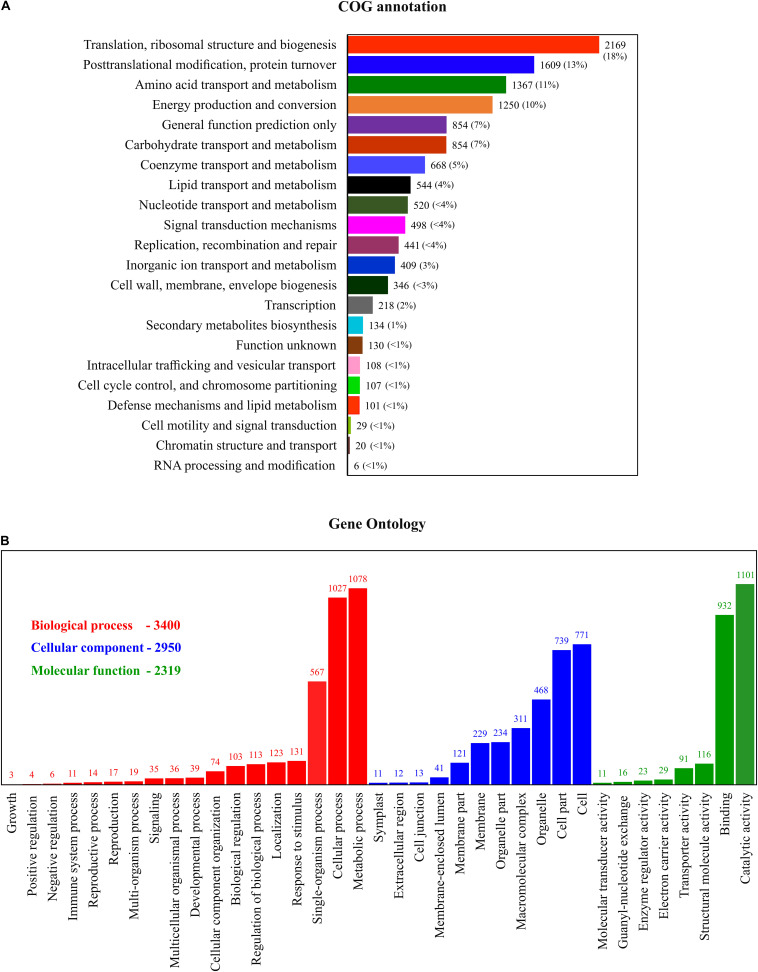
Functional annotation of the COG **(A)** and GO **(B)** analysis of the proteins identified in this study.

**FIGURE 5:**
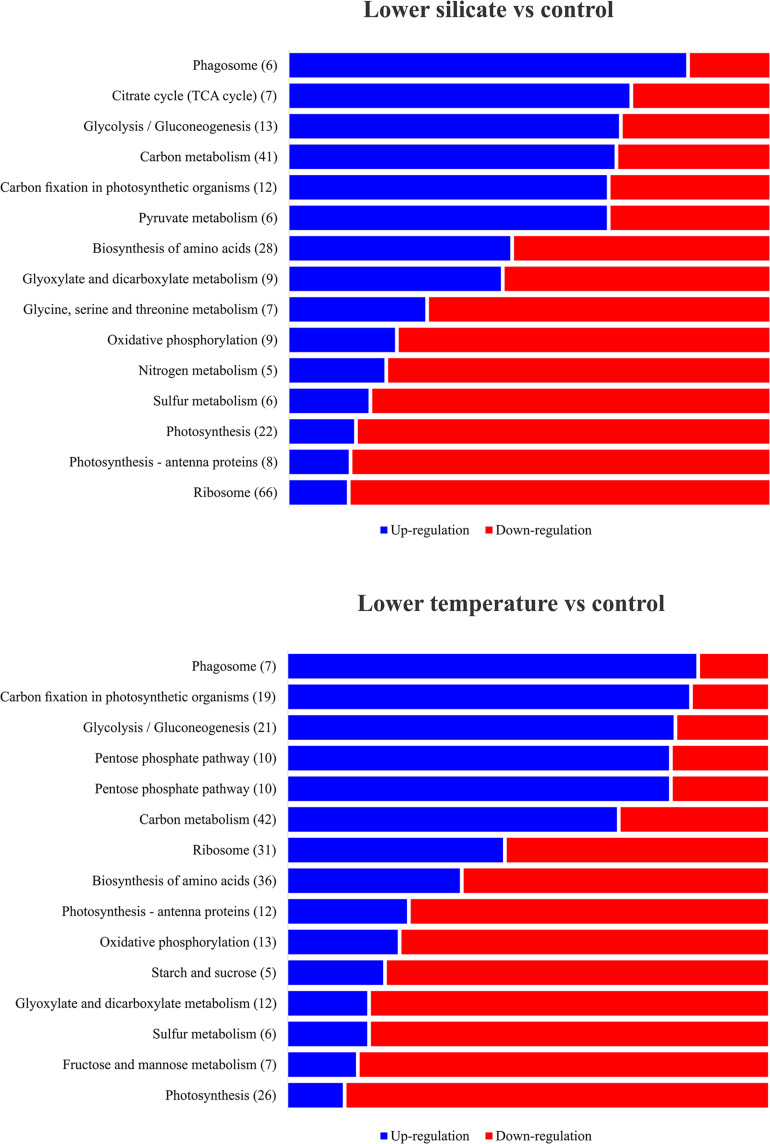
Most represented KEGG pathway enrichment analysis of differentially expressed proteins in the diatom *S. dohrnii*; those significantly enriched (*p* < 0.05) under lower-silicate and lower-temperature cells were denoted as upregulated (blue) and downregulated (red) within each pathway.

In total, 12,378 unigenes were identified in the COG database; they were attributable to 22 categories, based on sequence homology (Figure 4A). The largest group included proteins involved in ribosomes and their biogenesis (18%), followed by protein involved in post-transitional modification, protein turnover and chaperones (13%), and amino acid transport and metabolism (11%). To further understand the specific functions of these unigenes, they were subject to GO analysis; they were classified into three ontologies and 37 sub-categories (Figure 4B). The most represented ontology in molecular function was a catalytic activity, associated with photosynthetic electron transport. In terms of structural components, proteins involved in the cell cyclic, photosynthetic membranes and chloroplast thylakoid membrane are the most represented. For the biological process, a large portion of the protein identified is related to metabolic regulations of ATP synthesis, proton transport, and glycolysis. To understand the DEP involvement into the specific biological pathway, KEGG pathway enrichment analysis was carried out and shows (Figure 5) the most represented pathways in both silicate- and temperature-limited cells. Pathways of photosynthesis, light-harvesting, oxidative phosphorylation, carbon fixation, carbon metabolism, amino acid biosynthesis, and ribosome metabolism were involved in most DEPs in both lower-silicate and lower-temperature cells, although the number of proteins and fold change in each pathway is different.

### Changes in the Nutrients Transport

In lower-silicate cells, two silicon transporters (SIT1 and SIT2) were upregulated and 10 nitrate transporters, including Nitrilase (NIT2), NRT2, Urease (URE), and glutamate dehydrogenase, were downregulated (Table 1). In cells grown at lower temperatures, three transporters associated with nitrate assimilation, i.e., nitrate transporter (NRT1), urea protein transporter (DUR3), and xanthine uracil permease (TN.NCS2), and phosphate transporters [vacuolar transporter chaperone 4 (VTC4), and 5′-nucleotidase (ushA)] were downregulated. In addition, fewer sulfate transporters, methionine S-adenosyl transferase, and sulfate transporters were also noticed being downregulated in cells treated at lower temperatures; however, this has not been differentially expressed in cells treated with lower silicate.

**TABLE 1:** Differentially abundant proteins/transporters associated with the utilization of silicon, nitrate, phosphate, and sulfate in *S. dohrnii* treated with lower silicate and temperature.

**Transporter Name**	**Accession ID**	**Unique peptides**	**Fold change**
**Lower silicate vs. control**			
**Silicate utilization**			
Silicic acid transporter (SIT1)	XP_002290700.1	3	4.23
Silicic acid transporter (SIT2)	XP_002295920.1	5	3.41
**Nitrate utilization**			
Nitrate/nitrite transporter (NRT1)	AAV67002.1	6	–1.26
Nitrate/nitrite transporter (NRT2)	EJK46860.1	3	–1.67
Glutamate dehydrogenase	XP_002289225.1	2	–0.82
Aliphatic amidase	XP_002289996.1	3	–1.22
Nitrilase (NIT2)	XP_002290043.1	4	–0.91
AMP deaminase	XP_002289781.1	6	–1.41
5-hydroxyisourate hydrolase	XP_002288652.1	11	–1.19
Allantoicase	XP_002289615.1	2	–1.81
NADPH Nitrite reductase	XP_002287665.1	13	–1.14
Urease (URE)	XP_002296690.1	9	–1.23
**Lower temperature vs. control**			
**Nitrate utilization**			
Nitrate/nitrite transporter (NRT1)	EED92802.1	4	–0.68
3 Urea-proton symporters (DUR3)	XP_002292926.1	4	–1.71
Xanthine/uracil permease (TN. NCS2)	EED96094.1	7	–1.14
**Phosphate utilization**			
Vacuolar transporter chaperone 4 (VTC4)	EED87388.1	5	–1.43
Phospholipase D1/2 (PLD1_2)	XP_002288407.1	3	–2.17
5’-nucleotidase	XP_002295180.1	2	–1.87
Betaine aldehyde dehydrogenase	ACI64514.1	11	–1.31
**Sulfur utilization**			
Phosphoadenosine-phosphosulfate reductase	EED88796.1	6	–1.39
Methionine S-adenosyl transferase	BAH30220.1	6	–2.24
Sulfate transporter	XP_002286457.1	2	–1.84

*Detailed annotation of these nutrient transporters is given in Supplementary Table 2.*

### Changes in the Photosynthesis Metabolism

In this study, 20 downregulated proteins associated with photosynthesis were identified in the Si-limited treatment and 23 were identified in the lower-temperature treatment (Supplementary Table 3). Of these downregulated proteins, six and eight proteins specifically responded to silicate and temperature limitations, whereas 14 proteins responded commonly at both stress conditions. Downregulated proteins were classified into five groups: Photosystem II (PSII), Photosystem I (PSI), Light-harvesting complexes (LHC), Photosynthetic electron transport, and Chloroplast F-type ATPase. Quantitative proteomics revealed that six PSII proteins (*Psb A, B, C, D, E, H*), two PSI proteins (*Pas A, B*), six LHC proteins, and four F-type ATPase proteins were downregulated in lower Si-grown cells, whereas in lower-temperature cells, four PSII proteins (*Psb B, E, D, H*), two cytochrome proteins, one protein in PSI, and 10 LHCs were downregulated. Though many of the proteins were affected by both treatments, the extent of the regulation (fold change) varied: lower silicate appears to have a stronger impact on the PSII complex proteins, while the lower temperature had more influence on the LHC proteins.

### Changes in the Carbon Fixation and Carbohydrate Metabolism

In total, 41 proteins involved in carbon metabolism were differentially expressed in the lower Si treatment; in cells acclimated to lower temperature, 42 proteins showed changes in abundance relative to control (Supplementary Table 3). Of this DEP, 22 proteins were specifically expressed in lower-silicate cells, whereas 21 proteins were expressed in cells treated with lower temperature; 20 common DEPs were identified in both stress conditions. In cells treated with lower silicate, an important enzyme of glyceraldehyde-3-phosphate dehydrogenase (GAPD) was downregulated, while this protein was upregulated in cells treated at lower temperatures. Further, fumarate and pyruvate kinase (PYK) were upregulated in lower Si-grown cells, while these proteins in temperature-limited cells did not express differentially. In lower-temperature cells, phosphofructokinase (pfkA) was also upregulated, and so were some proteins related to pentose phosphate pathway (xylulose, sedoheptulose, and erythrose), but these genes were not expressed differentially in cells exposed to lower silicate. In addition, the specific response of lower temperature caused downregulation of oxaloacetate (OAA), and upregulation of sulfotransferase (ST), whereas these proteins (OAA) and ST abundances were up- and downregulated, respectively, in lower-silicate cells.

Some of the proteins involved in CO_2_ fixation were downregulated at both stress conditions, such as carbonic anhydrase (CA), ribulose-1,5-bisphosphate carboxylase (rbcL), phosphoenolpyruvate carboxylase (PEPC), and pyruvate phosphate dikinase (PPDK). Similarly, transketolase (TST), methionine chain elongation, (BCAT4), and S-adenosylmethionine synthetase (SAM) were downregulated at both stress conditions. Notably, key genes involved in lipid biosynthesis, i.e., acetyl-CoA carboxylase (ACACA), and long-chain fatty acyl-CoA were upregulated along with isocitrate (ICDH), pyruvate dehydrogenase (PYD), and succinate (SDH). Moreover, many proteins involved in glycolysis, TCA, and amino acid biosynthesis—phosphoglycerate mutase (PGM), glucose-6-phosphate isomerase (GPI), phosphoglycerate kinase (PGK), pyruvate dehydrogenase (PDC), fructose-bisphosphate aldolase (FBA), and citrate synthase (CS)—were similarly expressed in both stress conditions, although to a different extent.

### Changes in the Ribosome Metabolism

In total, 66 ribosomal proteins consisting of 47 large subunit and 19 small subunit proteins were differentially expressed in cells cultured with lower silicate (Supplementary Table 3). Among them, 45 proteins in large subunits and 16 proteins in small subunits were downregulated. Of these downregulated proteins, 10 were proteins associated with chloroplast in the large subunit and 4 were proteins related to 40S and 60S in the small subunit. Similarly, in lower-temperature cells, 31 proteins were differentially expressed, among them 15 proteins in large subunits and 3 proteins in small subunits were downregulated. Notably, 47 proteins were specifically expressed in lower-silicate cells, 19 proteins were expressed in cells treated at lower temperatures, whereas 22 proteins were commonly expressed in both stress conditions, although to a different extent.

### Changes in the Cell Cycle and Nucleus Related Proteins

In this study, many proteins related to cell cycle and nucleus were identified, and most of them were being downregulated. In lower Si treatment, a total of 49 proteins related (r, m, and t-RNA) to the ribosome, transporting coding sequences and translating information to protein, were downregulated (Supplementary Table 2). Cells exposed to lower temperatures downregulated 29 proteins related to DNA binding and its transcription to RNA (Supplementary Table 2). Specifically, high light-induced proteins and cullin protein were downregulated in cells acclimated to lower temperature. Similarly, dead box RNA helicase and GTP proteins were downregulated in the Si-deficient cells. An important cell cycle control protein, casein kinase II, was downregulated in low Si, while it was not expressed in lower-temperature cells. In lower-temperature cells, a core histone (H3) and one variant (H2A) associated with DNA binding and histone arginine N-methyltransferase were downregulated; in the Si treatment, histones (H3, H4) and histone deacetylase were downregulated. Proliferating cell nuclear antigen (PCNA), cyclin-dependent kinase (CDK), and mismatch repair (MMR) protein were decreased in both Si- and temperature-limited cells with varied downregulation ratios.

## Discussion

### Specific Cellular Nutrient Assimilation

In general, cellular functional efficiency relies on nutrient transporters, which define what metabolites and compounds can across the membrane. In this study, changes in the Si concentration and temperature availability can regulated not only nutrient transporters (Table 1) but also the utilization of organic nutrients (Figure 1). Recent transcriptome and proteomic studies on diatoms have shown various SIT upregulation during the silicon limitation (Shrestha et al., 2012; Du et al., 2014; Smith et al., 2016; Brembu et al., 2017). The outcome of these studies has shown that during silicon limitation, SITs act as a transcriptional cascade with encoding unknown amino acids to facilitate the silicic acid transport. Simultaneously, these encoded SITs have a unique subfunction in diatom metabolism, preventing translation into full-length protein and cell cycle arrest (Shrestha and Hildebrand, 2015; Durkin et al., 2016). These investigations have concluded that during silicon limitation, SITs have interrelation with cytoplasm to facilitate silicic acid transport, with the simultaneous improper binding of other functional proteins.

Nitrate is often mentioned to limit diatom growth in the ocean because of its contribution to binding chlorophyll, amino acids, and nucleic acids. Earlier proteomic observation on diatoms showed that limitation of iron, phosphate, and silicate has a coupling effect with N transporters, leading to downregulated nitrate transporters and their assimilation (Nunn et al., 2013; Chen et al., 2018). Similarly, many downregulations of nitrate transporters observed in this study responded to Si deficiency (Table 1), indicating lower nitrate assimilation (Figure 1) and cellular N quota (Figure 1). In any form of cell, extracellular N must convert into ammonium before assimilation into amino acids or other nitrogenous compounds. In this study, decreased abundance of ammonium converted transporters URE and AMP deaminase in Si-limited cells, suggesting a possible reduction of ammonium conversation and therefore N utilization. Proteins of Nitrilase (NIT2) and Aliphatic amidase play an essential role in N utilization (Howden and Preston, 2009), and downregulation of these transporters in Si-deficient cells also supports the reduction of N assimilation. In addition, allantoicase and 5-hydroxyisourate hydrolase involved in the purine utilization were also being downregulated in Si-limited cells, which is a substitute of N source for diatoms during stress conditions, and could regulate cellular development (Alipanah et al., 2015). Besides, decreased abundances of glutamate dehydrogenase under Si-limited conditions also led to decreased glutamate synthase, which has a critical role in intracellular N flow, as both N accepter and donor (Miflin and Habash, 2002). It is clear that *S. dohrnii* responded to lower Si caused and limited the N assimilation and utilization for the associated cellular process.

Similar to nitrate, phosphate also essential for amino acid and nucleic acid binding; in the ocean, polyphosphate is thought to be the product of phosphate storage in diatoms (Martin et al., 2011; Dyhrman et al., 2012). Accordingly, proteins containing polyphosphate synthase subunit (VTC4) are essential for maintaining intracellular phosphate homeostasis (Secco et al., 2012). In this study, cells treated at lower temperatures caused downregulation of VTC4 protein, suggesting that *S. dohrnii* was unable to store phosphate to fulfill its intracellular requirement. This is consistent with transcriptome (Zhang et al., 2016) and proteome response (Dyhrman et al., 2012) of diatoms *Skeletonema costatum* and *Thalassiosira pseudonana* to changing phosphate conditions. Diatoms can replace phospholipid with sulfur-containing (sulfolipids) and nitrogen-containing (betaine lipids) ones during phosphate deficiency to reduce the intracellular phosphate requirement of cells (Van Mooy et al., 2009). Downregulation of these transporters in lower-temperature cells suggests that *S. dohrnii* was unable to utilize non-phosphate-containing lipids and therefore affects the phosphate-associated cellular process. This finding is consistent with the earlier response of diatoms *S. costatum* (Zhang et al., 2016), *T. pseudonana* (Dyhrman et al., 2012), and *Chaetoceros affins* (Van Mooy et al., 2009) to changing phosphate conditions. The results on intracellular phosphate content (Figure 2) and nutrient concentration (Figure 1) in lower-temperature cells supported this speculation. In addition, the lower temperature also caused downregulation of three sulfur transporters, indicating the inadequate synthesized amount of cysteine, methionine, and glutathione with ultimate impact of growth rate and cellular development of diatom (Takahashi et al., 2011).

### Specific Cellular Response in Photosynthesis and Associated Metabolism

**FIGURE 6:**
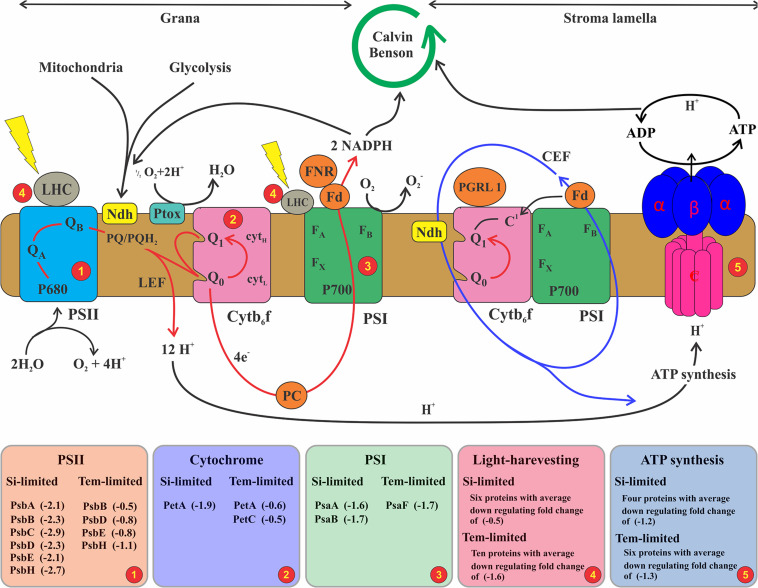
Schematic illustrating the photosynthetic electron transport chain and state transition model. The depicted model consisting of linear and cyclic electron flow and ATP generation. Linear electron flow (red arrow) associated with photosystem II (PSII), cytochrome b6f (Cytb6f), photosystem I (PSI), and ferredoxin-NADPH (FNR). PSII is composed of activation of oxidoreductase transferring an electron from water to plastoquinone, resulting in the reduced form of PQH_2_. In this process, when adequate linear electron transfer occurs, it is catalyzed by FNR to NADPH on one side, and simultaneously, proton gradient drives the ATP synthase (ATPase). Cycling electron transport (CEF) (blue lines) is represented by a single pathway involving ferredoxin-PQ reductase, which transfers electrons from Fd to PQ. Further, under aerobic conditions, the plastoquinone terminal (PTOX) could oxidize the PQ pool; therefore, electrons, e.g., from glycolysis, are imported into the chloroplast and lead to a reduction in the PQ pool by an NADH dehydrogenase (Ndh). In this study, the net production of electron conversation by PSII was decreased over a twofold change because of downregulated PSII primary proteins that catalyze this process (see Supplementary Table 3). Subsequently, less electron and proton were pumped to the PSI to NADPH and ATP synthase for the Calvin cycle and carbon fixation process. Higher downregulation in LHC 1 by lower temperature (see Supplementary Table 3) affects the CEF electron, which has been sent to ATP synthase with less ATP formation for other cellular processes depending on these energies.

Nitrate and phosphate are important for many physiological processes, including essential elements for protein synthesis. Reduction of these two nutrients’ assimilation could cause a decreased synthesis rate of photosynthetic proteins (Bucciarelli and Sunda, 2003). Accordingly, in cells treated with lower silicate, PsbA, PsbB, PsbC, and PsbD proteins were downregulated, with those being assembled in photosystem II acting as a reaction center with complex light-harvesting also involved in the production and transfer of linear electron flow for the NADPH production (Figure 6). In cells grown under Si-deficient conditions, these proteins were downregulated > 2-fold, whereas in lower-temperature treatment, it decreased < 1-fold (Supplementary Table 3), indicating higher influences on the PSII complex and associated changes in the linear electron flow by Si limitation than temperature. Similar findings were seen in the proteome level on diatoms responding to iron (Nunn et al., 2013), nitrate (Hockin et al., 2012), and silicate limitation (Thangaraj et al., 2019), showing that downregulation of PSII proteins leads to impact the linear electron transfer and consequent oxidation of PSI complex and NADPH production.

Despite chlorophyll and fucoxanthin being rich with nitrogenous compounds, lower temperature regulated higher LHC and PSI proteins with greater decreases in protein abundances compared to Si limitation (Supplementary Table 3), suggesting that light-capturing capabilities were compromised with lower temperature. PSI and LHC proteins are involved in binding chlorophyll and catalyze light-induced photochemical processes. For example, PsbF is involved in increasing electron transfers and iron-binding processes, while fucoxanthin and chlorophyll proteins encompass the light-harvesting processes. In this study, decreased abundance of these proteins in lower-temperature cells may cause regulation in the cyclic electron flow (Figure 6), leading to redox imbalance in photosynthesis metabolism. Non-photochemical quenching (NPQ) and alternative electron transport (AEF) have been carried out by algae to overcome the redox imbalance. NPQ is an important photo-protective process that dissipates excess energy during light changing conditions (Niyogi, 2000). To avoid energetically costly damage to the cell, diatoms have evolved a series of defense mechanisms that are controlled at the metabolomic level. For instance, *Phaeodactylum* has a high capacity for the NPQ during temperature fluctuation (Lavaud et al., 2002) through xanthophyll pigment cycling (Goss and Lepetit, 2015) and the light-harvesting proteins (Bailleul et al., 2010). Further, in higher plants, the PSII protein PsbS proved to be an important component for NPQ (Johnson and Ruban, 2010), but no homolog for this gene has been identified in diatoms (Armbrust et al., 2004; Bowler et al., 2008). However, several antenna proteins in diatoms might serve the role of PsbS in photoprotection (Zhu and Green, 2010), suggesting that there might be an interconnection between PSI and LHC to be involved in NPQ of *S. dohrnii* in this study under lower temperature.

AET pathways remove excess reductant from thylakoid membranes (Peltier et al., 2010) to consume excess reducing power generated by photosynthesis, thus decreasing the probability of ROS formation (Niyogi, 2000). The proportion of electrons consumed by AET, measured as light-dependent oxygen consumption, changes with changing growth in *T. pseudonana*, suggesting that AET can also be an important component for maintaining redox balance (Waring et al., 2010) in this study. PSII associated with electron flow, a plastid terminal oxidase, and the transfer of reduced carbon compounds to the mitochondria for oxidation, or the Mehler reaction could be the mechanism of AET in diatoms. AET including the Mehler reaction can consume up to 50% of the electrons released by PSII in diatoms (Waring et al., 2010). Similarly, an earlier study on *Phaeodactylum* photo physiology under changing environmental conditions predicted that AET could represent an important proportion of the total electron transport in thylakoids (Wagner et al., 2006). Altogether, it appears likely that AET significantly contributed to photoprotection in our experiments, especially Si limitation cells, where PSII proteins decreased dynamically at lower temperatures.

In this study, we did not measure direct AET activities but identified proteins that were involved in this metabolism and were differentially expressed in Si- and temperature-limited cells. For example, NADH dehydrogenase and alternative oxidase (AOX) were downregulated in cells that grew with lower Si, while in lower-temperature cells, these proteins were upregulated. This finding was similar to Thangaraj et al.’s results, in which downregulation of NADH dehydrogenase and AOX response to Si limitation of the diatom *S. dohrnii* were revealed. The NADH dehydrogenase catalyzes oxidation of NADH to NAD^+^ and transfers electron to ubiquinone. Upregulation of this NADH dehydrogenase could accelerate the rate of electron transport in the respiratory chain, while downregulation led to decrease its process. The mitochondrial AOX protein used for removing an excess electron in the nutrient-limited diatom (Allen et al., 2008) and upregulation of this protein could be involved in the mitigation of mitochondrial ROS production (Lin et al., 2017) in temperature-limited cells, whereas downregulation of this AOX protein and many other essential enzymes of the FoF1-type proteins in *S. dohrnii* suggests the regulation of ATP production with a coincident blockage of the respiratory chain under Si limitation. Consistent proteomic regulation was seen earlier on diatoms and cyanobacteria responding to a lower temperature (Mackey et al., 2013; Dong et al., 2016) and Si concentration (Chen et al., 2018; Thangaraj et al., 2019). Taken together, in *S. dohrnii* PSII, linear electron flow (grana) to NADPH mechanisms could be regulated by (Si) concentration, whereas PSI, LHC, and cyclic electron flow (stroma) to ATP metabolisms could be modulated by temperature limitation.

### Specific Cellular Response in Carbon Metabolism

**FIGURE 7:**
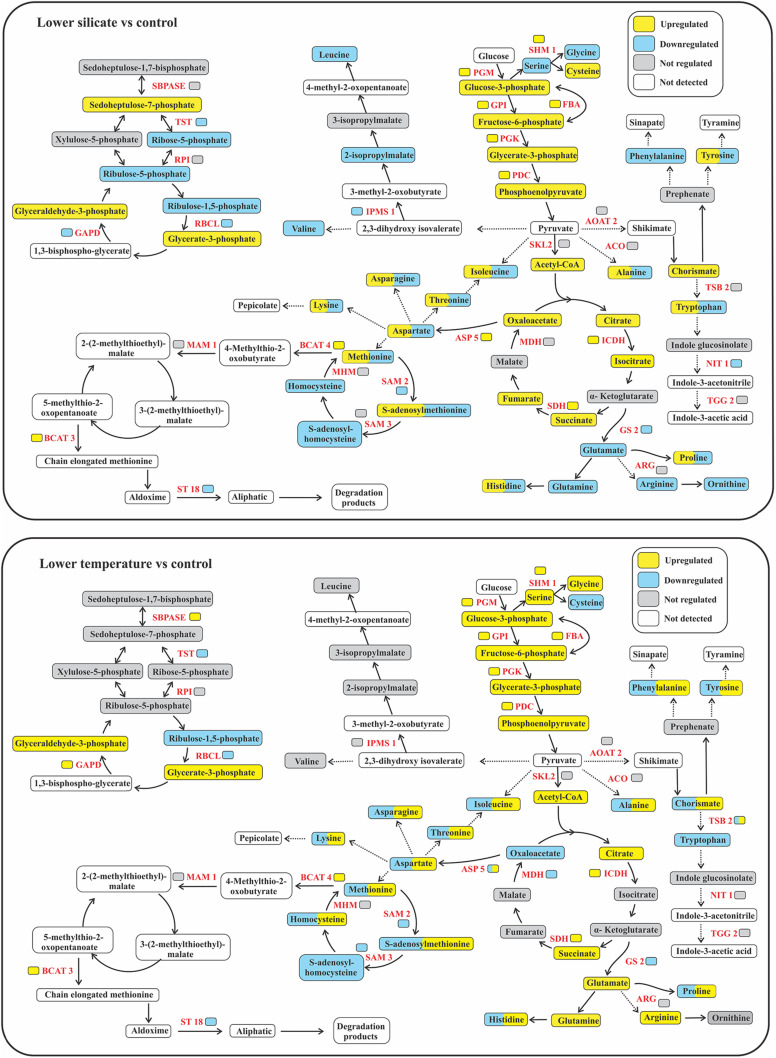
Changes in the protein abundances and carbon flow in the central carbon metabolism of the response of the diatom *S. dohrnii* to silicate and temperature limitation. SBPASE, Sedoheptulose-1,7-bisphosphatase; RPI, ribulose-5-phosphate isomerase; TST, transketolase; RBCL, ribulose bisphosphate carboxylase large chain; GAPD, glyceraldehyde-3-phosphate dehydrogenase; PGM, phosphoglucomutase; SHM, serine hydroxy-methyltransferase; GPI, glucose-6-phosphate isomerase; PGK, phosphoglycerate kinase; FBA, fructose-bisphosphate aldolase; PDC, pyruvate dehydrogenase; AOAT2, alanine-2-oxoglutarate aminotransferase; SKL, shikimate kinase; ACO, aconitate hydratase; ICDH, isocitrate dehydrogenase; SDH, succinate dehydrogenase; MDH, malate dehydrogenase; ASP5, aspartate aminotransferase 5; SAM2, S-adenosylmethionine synthetase 2; SAM3, S-adenosylmethionine synthetase 3; MHM, 5-methyl-tetra hydropteroyltriglutamate-homocysteine methyltransferase; BCAT4, branched-chain amino acid transaminase 4; MAM, methylthioalkylmalate synthase; BCAT3, branched-chain amino acid transaminase 3; IPMS, 2-isopropylmalate synthase; ST18, sulfotransferase 18; TSB2, tryptophan synthase beta-subunit; NIT, nitrilase; TGG2, thioglucoside glucohydrolase 2; GS2, glutamine synthetase.

The carbon and carbohydrate metabolism in diatoms involves complex enzymatic steps or metabolic reactions to convert carbohydrates into metabolic precursors (Figure 7) for cellular development; changes in those enzymes could impact the fundamental catalytic information among its intracellular process. In this study, a vital enzyme of GAPD was downregulated in Si-limited cells, while this gene in cells grown at a lower temperature was upregulated. GAPD is a major consumer of NADPH in diatoms; this allows for the redirecting of NADPH to other reductive cellular processes for the ecological success of diatoms under stressful conditions (Mekhalfi et al., 2014). Fumarate and PYK proteins were upregulated in cells grown with lower Si, while these proteins did not change their abundances in lower-temperature cells. Fumarate involves the major steps of the TCA cycle, which can modify protein binding and enzymatic activity (Leshets et al., 2018), while PYK is involved in the lipid biosynthesis of diatoms (Ma et al., 2014). Upregulation of these fumarate and PYK in this study was consistent with their variation in the transcriptome (Bender et al., 2014) and proteome (Smith et al., 2016) response to nitrate and iron deficiency. Further, important amino acids arginine, glutamate, and glutamine were downregulated in Si-limited cells, but they were upregulated in cells with lower temperature. In diatoms, the joint reactions of these enzymes are the primary route of ammonium assimilation and other nitrogenous compounds, providing an important link between ammonium assimilation and carbon metabolism. These results suggested limited nitrogenous assimilation in Si-limited cells compared with enhanced assimilation in lower-temperature cells, supporting the physiological data of inorganic nutrients (Figure 1).

Similar to Si limitation, *S. dohrnii* grown at lower temperatures also show distinct proteomic regulation in carbon metabolism. For example, a major intermediate of TCA cycle oxaloacetate (OAA) was downregulated in cells grown at lower temperatures while this protein was upregulated under silicate deficiency. Downregulation of this OAA could inhibit the partial mitochondrial function and the production of precursors for important biomolecules. Further, the key enzyme of phosphofructokinase (pfkA) and PPP proteins (xylulose, sedoheptulose, and erythrose) were upregulated in lower-temperature cells, while these genes did not express in lower-Si cells. The pfka catalyzes the irreversible step in glycolysis, and therefore, upregulation of this enzyme under stress could significantly enhance the glycolytic pathway in diatoms (Yang et al., 2014). Changes in the PPP proteins can operate in different modes: (i) ATP-consuming mode (where there is no loss in carbon or net production of ribulose-5-phosphate as building blocks for other molecules such as AA), (ii) NADPH-producing mode (where all carbon from G-6-P is released as CO2), or (iii) PPP coupled with glycolysis to produce both ATP and NAD(P)H and CO2. These various modes interact and equilibrate with each other when there are energetic needs for the cell, which could favor cellular functions of protein binding and cell cycle regulation under stress conditions. Amino acids of serine and glycine are important for the diatom cell wall formation (Hecky et al., 1973) and were upregulated in lower-temperature cells, while these metabolisms were downregulated in Si-limited cells.

### Changes in the Ribosome and Its Associated Metabolism

In general, the aminoacyl-tRNA delivers the amino acid to the ribosome for incorporation into the polypeptide chain and functional proteins that are being produced during the translation process. If the incorrect amino acid is attached during this process, then the tRNA is improperly charged and sends improper amino acids to the ribosome via a translation mechanism that would dynamically impact the ribosome complex and its function. In this study, 49 proteins related to (r, m, and t-RNA) translation mechanisms were downregulated in lower-Si cells, indicating that this mechanism could be nutrient (Si)-dependent in *S. dohrnii*, resulting in 61 downregulated proteins in ribosome metabolism and reduced protein synthesis (Figure 2). On the contrary, 29 proteins related to DNA binding to RNA transcription were downregulated in lower-temperature cells, suggesting that this could be temperature-dependent, resulting in downregulation of only 18 ribosomal proteins. Similarly, recent reports also found that the temperature depended on the transcription processes in *Arabidopsis* (Cortijo et al., 2017) and other eukaryotes (Oliveira et al., 2016).

The primary role of the ribosome is to synthesize functional proteins that are needed for other metabolic functions. These protein synthesis and translation processes are tightly coordinated with cell growth and proliferation (Ruggero and Pandolfi, 2003). Impairment of any of these changes can severely alter cell growth and perturb organism development; this has been demonstrated in almost all eukaryotic organisms (Zhou et al., 2015). For example, a genetic study on yeast revealed that changes in the ribosome assembly result in a lesser amount of protein synthesis and cell size (Jorgensen et al., 2002). Similarly, increasing abundances of elevating ribosome proteins promote cell growth and proliferation by boosting protein synthesis (Van Riggelen et al., 2010). Likewise, several studies have demonstrated in a wide range of organisms that the efficiency of protein synthesis is governed by the rates of ribosome complexes and their components (Choi and Puglisi, 2017; Verma et al., 2019). However, in diatoms, the downregulation of the ribosome not only decreases the material and energy expenditure but also smoothens the polypeptide processing and proteasomes to facilitate the protein turnover under changing Si concentrations (Shrestha and Hildebrand, 2015) and temperatures (Liang et al., 2019). Such protein turnover in diatoms has been reported previously to decrease cell growth, development, proliferation, response to changes in temperature (Liang et al., 2019), CO_2_ (Beszteri et al., 2018), and nutrient stress condition (Dyhrman et al., 2012; Chen et al., 2018). These results indicate that if components of a ribosome are increased beyond normal, it would drive efficient protein synthesis, cell growth, and proliferation, while if it decreased, it could inhibit growth and proliferation. It was evident that decreased ribosomal complexes, protein synthesis (Figure 2), and cell growth (Figure 1) were present in both silicate and temperature limitation.

### Common Cellular Responses of *S. dohrnii* to the Lower Silicate and Temperature

In this study, proteins associated with photosynthesis, LHCs, carbon metabolism, and ribosomes were differentially expressed in both Si- and temperature-limited cells. Recent studies show that lower Si and temperature decreased a diatom’s protein synthesis and led to metabolic imbalances and oxidative stress with a negative impact on photosynthesis and carbon fixation (Shrestha et al., 2012; Dong et al., 2016; Thangaraj et al., 2019). Among PSII functional proteins, PsbB, PSbD, PsbE, and PsbH were downregulated at both stress conditions, indicating that these proteins are both nutrient (Si)- and temperature-dependent in the marine environment. Similarly, in PSI and cytochrome, PetA and PsbF proteins were also being downregulated at both stress conditions. PetA binds chlorophyll and catalyzes the light-induced photochemical process, while PsbF stimulates electron transfer or the heme-binding or iron-binding process, which could be influenced by both stress conditions. LHC proteins located in the photosystem complex and their increasing enzymatic modulation are dependent on proteins that are rich in N, while their biosynthesis process depends on temperature limitation. Accordingly, lower N acquisition in Si-limited cells and limited catalytic process due to lower temperature (Thangaraj and Sun, 2020a) could share many downregulating LHC proteins in common (Figure 6). These results are consistent with the response of model diatoms *T. pseudonana* (Chen et al., 2018) and *P. tricornutum* (Yang et al., 2014) to nutrient limitation, suggesting that these both stress Si and temperature conditions could worsen light reactions of *S. dohrnii.*

Carbon fixation proteins: Phosphoribulokinase (PPDK), CA, and RuBisCO were downregulated in both stressed cells (Figure 6), suggesting that both Si and temperature limitation could reduce the C4-like carbon concentrating pathway and decrease carbon fixation, influencing the carbon inside the cell to be adjusted to the match reductant supply (Allen et al., 2008). Similarly, S-adenosylmethionine synthetase (SAM) was downregulated at both conditions, which is a storage form of methionine (Bourgis et al., 1999) that is involved in over 40 metabolic functions/reactions associated with nucleic acids, lipids, proteins, and secondary metabolites (Bartlem et al., 2000). Therefore, changes in this SAM in cells grown at stressed conditions could inhibit cellular development and growth (Reintanz et al., 2001). Besides, TST is a key enzyme involved in sugar metabolism and was downregulated at both stress conditions. In algae, TST is located in the plastid membrane, where photosynthesis occurs (Teige et al., 1998); thus, changes in TST could impact the photosynthesis process (Henkes et al., 2001). Despite different fold changes, isocitrate (ICDH) and succinate (SDH) were also being downregulated in both conditions. ICDH is a vital enzyme in the two-step process of producing alpha-ketoglutarate and CO_2_ in diatoms (Alipanah et al., 2015), while SDH involved in the mitochondrial electron transport chain for transferring electrons from succinate to ubiquinone could be influenced by both stress conditions.

Chen et al. (2018) recently reported that, although diatom carbon fixation was decreased during stress conditions, cellular C and lipid content was increased due to the upregulation of the glycolytic metabolic pathway. Their findings are also supported in the present study: upregulation of glycolytic pathways (Figure 6) resulted in enhancement in cellular C and lipid accumulation under both stress conditions (Figure 2). In addition, under both conditions, acetyl-CoA carboxylase (ACACA) was upregulated, which is responsible for the production of long-chain acyl-CoA that can be utilized for the cellular lipid synthesis and variant subcellular localization to different anabolic and catabolic pathways (Mashek et al., 2007). Further, identified upregulated glycolytic proteins in both stress conditions may be the response to decreased production of NADPH and ATP from photosynthetic light reactions. Alternatively, increased sugar (glucose, sucrose, and fructose) content could suppress the expression of light-harvesting proteins (Martin et al., 2011) and light reactions (Chen et al., 2005). This is consistent with the observation of the negative relationship between the photosynthetic rate and carbohydrate content (Araya et al., 2010). It is likely that the downregulation of aliphatic glucosinolates causes increased carbohydrate accumulation and imbalances between light and dark reactions, which, in turn, eventually decreases photosynthesis and slow growth rate at both stress conditions.

In addition, both stress conditions caused upregulation of the methionine chain-elongation (BCAT) protein, catalyzing the terminal steps of methionine, which could be a substrate enhancement for cellular development. In connection to this, S-adenosylmethionine was downregulated, suggesting that channeling methionine was inhibited to form SAM for one-carbon metabolism when this methionine pathway was regulated by both conditions. The result agrees with the mutation of SAM that can result in a dramatic accumulation of methionine (Shen et al., 2002) for cellular development. In addition, many amino acids in both conditions were expressed similarly to up- and downregulation, respectively. These included multiple aminotransferases, which yield fates for amino acids including the rearrangement of new amino acid formation or complete intracellular recycling to alpha, keto acids, ammonia, or pyruvate. The results show that, despite photosynthesis, electron transport, light-harvesting, and the carbon fixation processes being decreased by both conditions, the total lipid and cellular carbon content per cell increased due to the utilization of upregulated proteins in glycolytic pathways and long-chain fatty acids.

Histones are a primary component of eukaryotic chromatin; modulation of this can affect the DNA binding, replication, and protein folding relevant to cell cycle progress (Li et al., 2018). It is shown that decreased abundances of histone in both conditions in this study would greatly impact smooth cell cycle progress and cellular development of diatoms. The MMR protein and associated DNA repair are essential to maintain the integrity and continuity of general information in eukaryotic cells. The study shows downregulation of this MMR at both stress conditions, suggesting a reduction in the DNA repair process. PCNA is a potential molecular marker for the phytoplankton metabolic process, and its higher expression ensures a smooth cell cycle (Zhang et al., 2019). Consistent with this, decreased abundances of this PCNA in this study indicate a further reduction in the cell cycle process of *S. dohrnii.* Cell division CDK is an engine of the cell cycle in phytoplankton and their DNA replication (Zhang et al., 2019). Downregulation of this CDK at both conditions shows that it might play an essential role in decreasing cell growth and abundances under stress conditions like Si and temperature.

## Conclusion

The results showed that the limitation of both silicate and temperature has common and specific metabolic responses to inhibit cellular development and growth of the diatom *S. dohrnii.* Nutrient assimilation and utilization were decreased at both conditions at different extent, reflected to impact PSII and NADPH production in silicate-limited cells and PSI and ATP production in lower-temperature cells. Notably, both stress depressed carbon fixation and photorespiration but simultaneously endorsed lipid and carbohydrate accumulation. Besides, both limitations have their own cell cycle-related protein alteration leading to metabolic imbalances in the transcription and translation process in temperature- and silicate-limited cells, respectively. Moreover, ribosome assembly in both limitations decreased its process, although in different extent, leading to reduced functional protein synthesis for the cellular development and growth of the diatom *S. dohrnii.* The integrative approach revealed previously unrecognized silicate and temperature that are dependent on mechanisms in growth and cellular development of marine diatoms, which could be valuable to understand how nutrients (Si) and the temperature are different for a diatom’s functional activity in the marine environment for their ecological success.

## Data Availability Statement

The raw mass spectrometry proteomics data and analysis file have been submitted to ProteomeXchange via PRIDE database (www.ebi.ac.uk/pride/archive/) with identifier PXD021705.

## Author Contributions

ST and JS designed the study. ST performed the laboratory experiment, carried out data analysis, and defined the manuscript content. MG reviewed and revised the manuscript. JS coordinated this investigation and provided guidance, funding, and facilities to perform the experiment. All authors contributed to the article and approved the submitted version.

## Conflict of Interest

The authors declare that the research was conducted in the absence of any commercial or financial relationships that could be construed as a potential conflict of interest.

## Supplementary Material

Supplementary Table 1 Annotation details of overall identified proteins.

Supplementary Table 2 Identification of down and up-regulated proteins in silicate and temperature limited conditions.

Supplementary Table 3 DEP associated with photosynthesis, carbon and ribosome metabolism in both silicate and temperature limited conditions.
